# Strain‐Induced Moiré Polarization Vortices in Twisted‐Multilayer WSe_2_

**DOI:** 10.1002/smll.202503363

**Published:** 2025-05-20

**Authors:** Jeroen J.M. Sangers, Abel Brokkelkamp, Sonia Conesa‐Boj

**Affiliations:** ^1^ Kavli Institute of Nanoscience Delft University of Technology Delft 2628 CJ The Netherlands

**Keywords:** in‐plane polarization, moiré superlattices, strain‐induced polarization textures, twisted multilayer WSe_2_

## Abstract

Moiré superlattices in 2D van der Waals (vdW) materials enable the engineering of local polarization textures and electrostatic potential landscapes. While polarization vortices are demonstrated in bilayer transition metal dichalcogenides (TMDs), their formation mechanisms in multilayers remain unclear. Here, it is shown that in multi‐twisted small‐angle multilayer WSe_2_, nanoscale strain fields, not twist alone, govern the emergence, and stability of polarization vortices. Using 4D scanning transmission electron microscopy (4D‐STEM) with an electron microscope pixel array detector (EMPAD), local electrostatic potential variations and strain distributions are spatially resolved with nanometer precision. It is found that vortex‐like polarization textures emerge exclusively in regions with significant nanoscale strain, revealing a direct interplay between lattice reconstruction and Moiré‐induced polarization textures in twisted multilayers. The findings establish strain as a key tuning parameter for Moiré‐induced polarization control, providing new pathways for strain‐engineered 2D vdW materials, chiral dipole textures, and next‐generation low‐power electronic and optoelectronic devices.

## Graphical abstract

This paper explores strain‐induced polarization vortices in twisted multilayer tungsten diselenide (WSe_2_). Using four‐dimensional scanning transmission electron microscopy (4D‐STEM) and advanced strain mapping, we reveal how nanoscale strain, rather than twist angle alone, governs vortex formation in Moiré superlattices. Our findings highlight strain as a key tuning parameter for polarization control, enabling novel functionalities in low‐power electronic and optoelectronic devices.

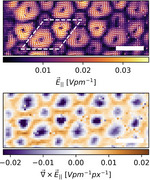

## 1 Introduction

The ability to control polarization in low‐dimensional materials has driven recent advances in quantum and nanoelectronics. Among these, 2D van der Waals (vdW) materials, such as transition metal dichalcogenides (TMDs), offer an ideal platform for designing tunable quantum systems.^[^ ^1^, ^2^ ^]^ The weak interlayer interactions in vdW materials allow for precise control over their stacking and twist angles,^[^ ^3^, ^4^ ^]^ leading to the formation of Moiré superlattices, emergent periodicities that enable the manipulation of electronic, polarization, and magnetic properties at the nanoscale.^[^ ^5^, ^6^, ^7^ ^]^

While bulk vdW materials are typically non‐polar, interfacial symmetry breaking, strain, and external fields can induce spontaneous electric polarization.^[^ ^8^, ^9^, ^10^, ^11^, ^12^ ^]^ Unlike conventional ferroelectrics, some vdW systems exhibit polarization textures that are highly tunable through stacking and strain, making them promising candidates for next‐generation flexible, low‐power, and reconfigurable electronic devices.

In twisted bilayer vdW systems, out‐of‐plane (OOP) polarization has been widely studied, with Moiré‐induced polarization emerging in materials such as hBN, MoS_2_, and GaSe, where stacking order governs polarization patterns.^[^ ^13^ ^]^ Furthermore, stacking registries defined by small twist angles induce Moiré‐driven polarization,^[^ ^14^, ^15^ ^]^ characterized by staggered polarization patterns with tunable magnitudes. Recent theoretical work suggests that marginally twisted bilayer TMDs can also host complex in‐plane (IP) polarization textures, including Bloch‐ and Néel‐type meron networks and vortex structures, arising from strain gradients and stacking configurations.^[^ ^16^, ^17^ ^]^ These topological polarization textures are expected to modulate band topology and domain dynamics, making them relevant for quantum materials applications.^[^ ^18^ ^]^

Despite this theoretical progress, the experimental validation of in‐plane polarization textures, particularly their dependence on strain, remains elusive. While investigations into twisted bilayers, such as MoS_2_, have revealed IP polarization vortices,^[^ ^15^ ^]^ the role of nanoscale strain in their formation remains poorly understood. A key challenge is the inherent difficulty in directly correlating structural configurations, strain fields, and in‐plane polarization properties at the nanoscale with sufficient spatial resolution. This necessitates advanced characterization techniques capable of resolving real‐space polarization textures with nanometer precision.

In this work, we investigate strain‐induced polarization vortices in twisted multilayer WSe_2_ using 4D scanning transmission electron microscopy (4D‐STEM) with an electron microscope pixel array detector (EMPAD). By resolving nanometer‐scale variations in local electrostatic potentials and strain fields, we demonstrate that while multiple regions exhibit small twist angles, polarization vortices emerge only in areas with significant nanoscale strain. This establishes strain, not twist alone, as the governing mechanism behind vortex formation, marking the first experimental validation of strain‐driven polarization textures in a multilayer Moiré system. Our findings reveal a direct interplay between Moiré superlattices, lattice reconstruction, and local electrostatic fields, positioning strain as a key tuning parameter for polarization engineering in vdW materials. These insights open pathways for strain‐tunable Moiré polarization control, with implications for low‐power electronics, topological excitations, and chiral dipole textures in 2D vdW materials.

## 2 Results

Twisted multilayer WSe_2_ specimens were fabricated using mechanical exfoliation and dry/wet transfer techniques (see Experimental for details).

**Figure 1:**
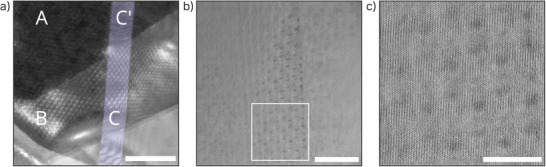
Structural characterization of twisted multilayer WSe_2_. a) High‐angle annular dark‐field scanning transmission electron microscopy (HAADF‐STEM) image of the twisted multilayer WSe_2_. The structure comprises three distinct regions formed by the stacking of three exfoliated flakes: region A (top left, darker contrast) is the bottom flake with 8 ML; region B (intermediate brightness) results from a 2 ML flake overlapping region A, forming a 10 ML double‐stacked area; and region C (highlighted in blue) is a second 2 ML flake that crosses both A and B, forming a ≈50 nm‐wide stripe that with a total of 12 MLs in the overlap. A Moiré pattern arises due to slight layer rotation misalignment during the stacking process, with the narrow strip further modulating the periodicity. b) High‐resolution transmission electron microscopy (HRTEM) image of the strip region in (a), revealing hexagonally symmetric Moiré patterns. c) Magnified HRTEM view of the region marked by a white square in **(**b). Scale bars: 100,, and 10 nm for (a), (b), and (c), respectively.

**Figure** **1**a presents a STEM high‐angle annular dark‐field (HAADF) image of the specimen, revealing three distinct regions labeled A, B, and C. Region A corresponds to the base 8 monolayers (ML) flake that extends beneath the entire sample. Region B is formed by the partial overlap of a second 2 ML flake on top of region A, resulting in a total thickness of 10 ML, refereed to as the double‐stacked zone. Region C denote a narrow stripe (highlighted in blue) approximately 50 nm wide and highlighted in blue, formed by a third 2 ML flake intersecting regions A and B, resulting in a local thickness of 12 ML, referred as the triple‐stacked stripe. These regions arise from the sequential stacking of three exfoliated WSe_2_ flakes. The number of monolayers in each region was confirmed by electron energy‐loss spectroscopy (EELS) mapping (see Experimental Section for details). The observed Moiré patterns arise due to rotational misalignment between the sequentially stacked flakes. The narrow triple‐stacked strip (region C) further modulates the periodicity by introducing additional twist angles relative to the underlying layers, resulting in overlapping Moiré patterns, as seen in Figure 1b. Fast Fourier Transform (FFT) analysis (Figure S1, Supporting Information) quantitatively confirms these rotational misalignments: the second flake (2 ML, forming region B) is rotated by 1.5 ° relative to the bottom flake (region A, 8 ML base), while the third flake (2 ML, forming region C) exhibits an additional misalignment of 2.3 ° relative to the base (A) and 1.0 ° relative to the underlying double‐stacked zone (B). An inverse FFT of the marked region in Figure 1b provides a magnified view of the selected region, revealing a hexagonal arrangement of dark contrast dots that further emphasize the periodicity of the Moiré superlattice.

### 2.1 Vortex Textures in Twisted Multilayer WSe_2_

In 2D van der Waals heterostructures, Moiré superlattices have been shown to induce emergent polarization fields due to local atomic registry variations. To investigate these effects at the nanoscale, we employ 4D‐STEM with an EMPAD detector,^[^ ^19^ ^]^ enabling a direct, real‐space mapping of local polarization and in‐plane (IP) (parallel to the specimen) electric fields. 4D‐STEM measurements provide momentum‐resolved diffraction datasets, capturing local structural distortions and electronic properties at the nanoscale.^[^ ^20^, ^21^ ^]^ From these datasets, the IP component of the local electric field in the twisted multilayer WSe_2_ system can be extracted.^[^ ^22^, ^23^ ^]^ To accurately resolve these IP electric field textures, the probe size must be carefully selected to ensure that 4D‐STEM measurements capture both long‐range Moiré effects and local atomic‐scale variations^[^ ^24^, ^25^ ^]^ (see Section S2, Supporting Information).

**Figure 2:**
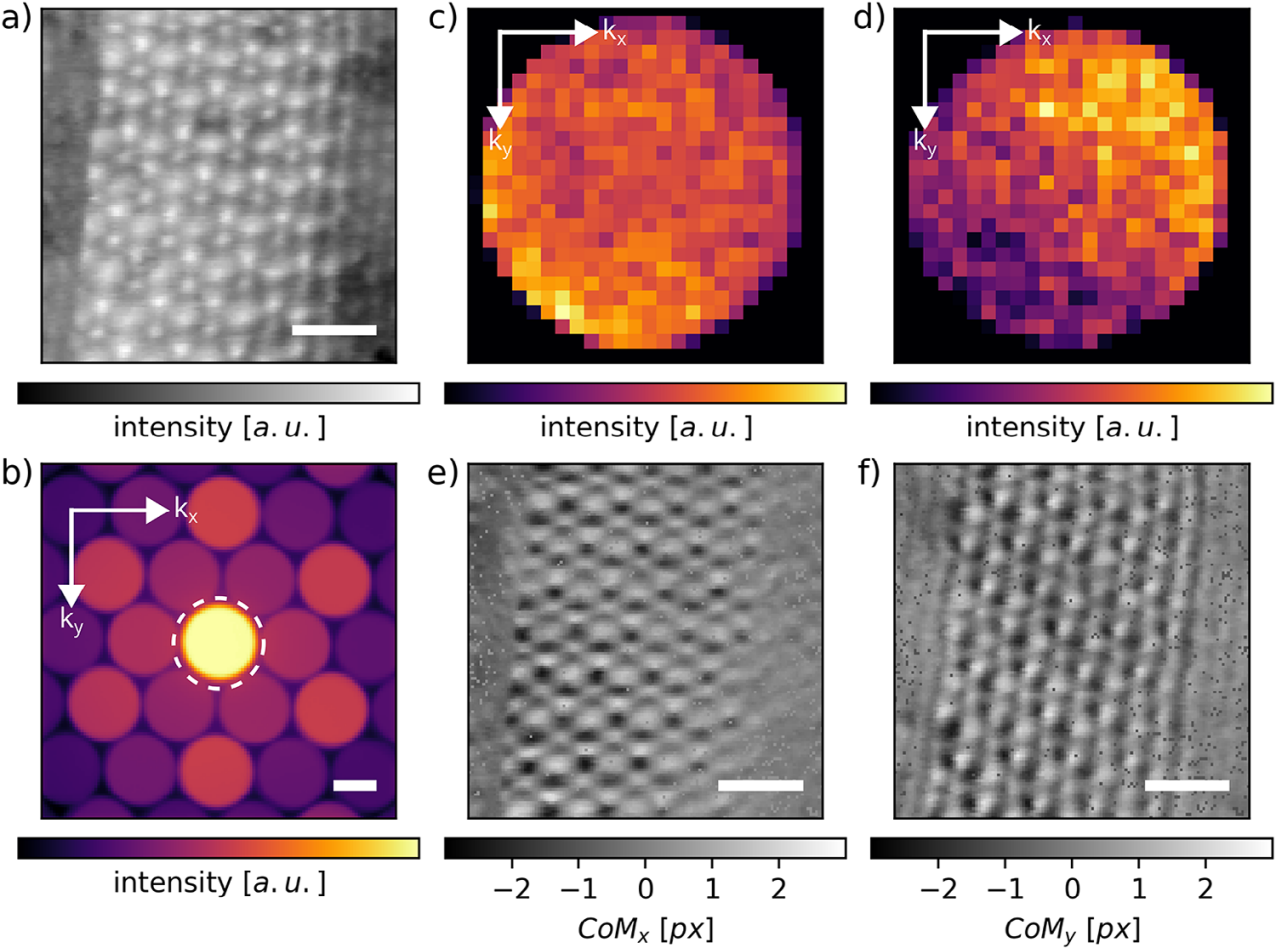
4D scanning transmission electron microscopy (4D‐STEM) analysis. a) Reconstructed annular dark‐field (ADF) image from the 4D‐STEM dataset, obtained by integrating the signal outside the bright‐field disk at each scan position. b) Representative electron diffractogram showing the intense centered bright‐field spot (dashed white circle) and undiffracted beam, whose center‐of‐mass (CoM) displacement is computed. Bragg spots outside the dashed circle contribute to the dark‐field signal in **(**a). c,d) Selected undiffracted bright‐field disks. **(**c) shows minimal intensity redistribution, whereas (d) shows significant redistribution. e,f) Maps of the *x*‐component (CoM_*x*_) and *y* −component (CoM_*y*_) of the center of mass, evaluated in the strip region of the twisted multilayer WSe_2_. Scale bars are 10 nm for (a,e,f) 2 n^−1^m for (b).

In 4D‐STEM, a convergent beam electron diffraction (CBED) pattern is recorded at each probe scan position, generating a complete 4D dataset consisting of spatially resolved diffraction patterns across the sample. From this 4D dataset, real‐space images such as the HAADF reconstruction can be obtained by integrating intensity outside the bright‐field disk at each scan position. A representative HAADF image, reconstructed from this dataset, is shown in **Figure** **2**a. Our analysis focuses on the overlapping region where the three structural domains intersect (white square in Figure 1b), where the Moiré pattern is mostly clearly defined, as well as the immediately adjacent double‐stacked regions on the left and right. This area includes a 1.5° between the bottom and middle flakes (regions A and B) (which appear with darker contrast in Figure 2a), and additional twists of 2.3° (A‐C) and 1.0° (B‐C) where the third flake (region C) overlaps the others, forming a locally modulated Moiré structure in the triple‐stacked stripe, as clearly seen in Figure 2a (bright central stripe). An example of CBED pattern, obtained with a 3.39 mrad convergence angle, is displayed in Figure 2b. The bright‐field disk, annotated with a dashed circle, exhibits the studied intensity redistribution. Two representative bright‐field disks with minimal and large intensity redistribution are plotted in Figure 2c,d, respectively. Integrating the intensity outside of the bright‐field disk provides a contrast signal used to reconstruct the image of the Moiré region (Figure 2a).

**Figure 3:**
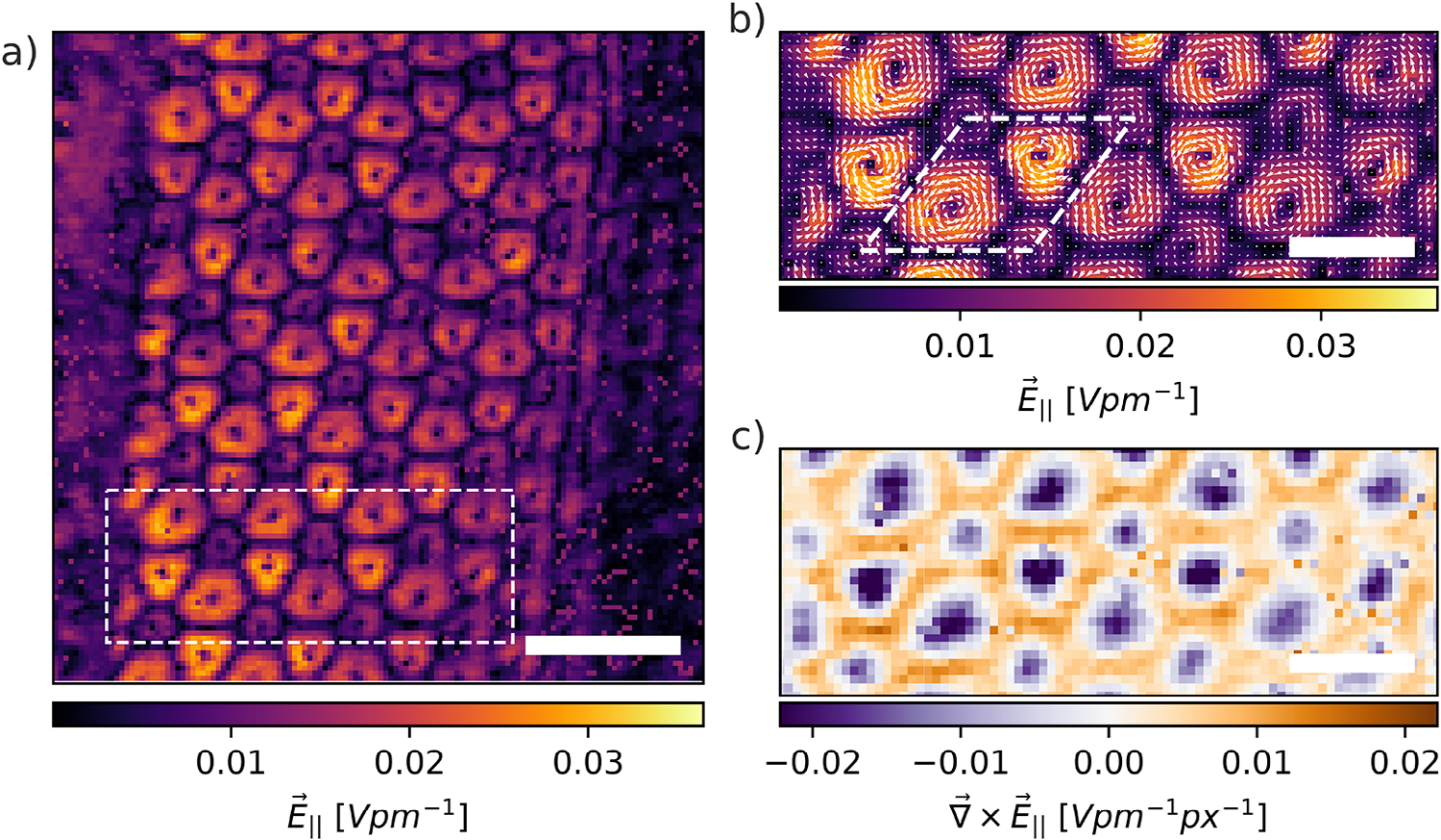
In‐plane electric field mapping from 4D‐STEM measurements. a) The in‐plane (IP) component of the local electric field ${\vec{E}}_{∥}$, obtained from the CoM maps in the same strip region. At each probe position, the strength and direction of the local electric field are displayed. b) Zoom‐in view of the region marked with a white dashed rectangle in (a). For illustration, the white dashed rhombus highlights the unit cell of the associated Moiré superlattice. c) The vorticity (curl) of the IP electric field displayed in (b). Negative vorticity values (strong counterclockwise rotation) and positive vorticity values (strong clockwise rotation) are plotted in purple and orange, respectively. Scale bars: 5 nm in all panels.

The local electrostatic potential variations were quantified through center‐of‐mass (CoM) analysis of the bright‐field disk intensity, extracted from the 4D‐STEM dataset.^[^ ^24^ ^]^ The CoM shift **k**_CoM_(**r**), corresponding to the integrated momentum transfer, was computed following established methods (see Experimental Section for details). Finally, the CoM distribution was evaluated across the specimen at each probe position **r** = (*x*, *y*), as shown in Figure 2e,f, which corresponds to the 2D maps of the intensity of the CoM_*X*_ and CoM_*Y*_ components, respectively. These CoM shifts are directly proportional to the momentum transfer experienced by the electron wave, allowing the calculation of the IP electric field components $\vec{E_{∥}}$ parallel to the specimen surface, given by: $\vec{E_{∥}}\left(\vec{r}\right)=-\left({\vec{k}}_{\mathrm{CoM}}\left(\vec{r}\right)\right)\frac{v}{e\cdot z}$, where *v* is the electron beam velocity, *e* is the elementary charge, and *z* is the sample thickness. Thus, the extracted CoM shifts provide a direct measure of the IP electric field across the specimen, which, in turn, is proportional to the local electric fields. Using this relationship, the spatial distribution of the IP electric field components ($\vec{E_{xy}}=\vec{E_{∥}}$) was reconstructed, as shown in **Figure** **3**a. The electric field vectors form a periodic, vortex‐like pattern that aligns with the underlying Moiré superlattice (Figure 3b), revealing the influence of the stacking registry on the electrostatic potential. Regions of higher electric field magnitude, highlighted in darker red, are concentrated in specific areas, spatially correlating with the high‐intensity contrast dots in the STEM‐HAADF image (Figure 1a.) This suggests a direct link between Moiré contrast and electrostatic modulation.

To further investigate these rotational field effects, we computed the vorticity of the IP electric field, $\nabla \times \vec{E}$, as shown in Figure 3c. Alternating orange (positive) and purple (negative) regions indicate clockwise and counterclockwise rotations of the electric field, respectively. The spatial periodicity of these vorticity features aligns with the electric field vortices observed in Figure 3b, confirming that the strongest electric field intensities coincide with regions of maximum rotational effects.

Interestingly, within the triple‐stacked stripe of this multilayer system, we observe a consistent vorticity in the same direction – counterclockwise – rather than alternating meron–antimeron textures as reported for bilayers systems.^[^ ^26^ ^]^ This suggests that the presence of a third overlapping flake in the triple‐stacked stripe influences the polarization topology, potentially stabilizing a uniform vorticity pattern. Interlayer interactions within this triple‐stacked region may enhance the alignment of polarization vectors across layers, thereby modifying the expected domain formation.

**Figure 4:**
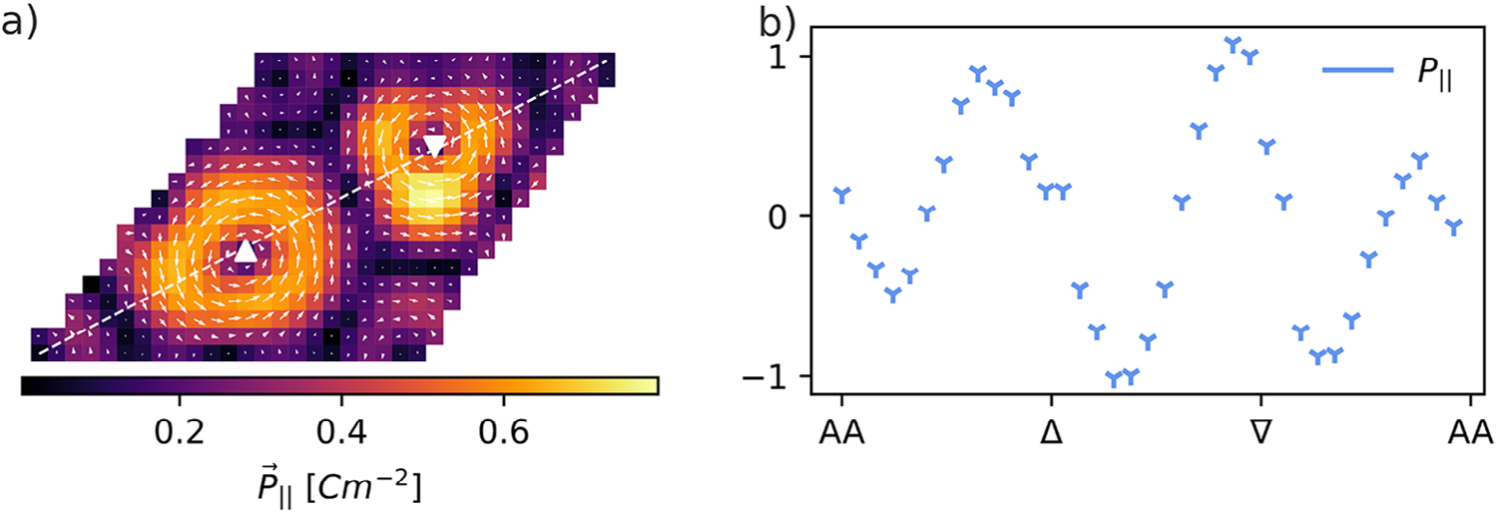
In‐plane polarization mapping of the Moiré unit cell. a) Nanoscale IP polarization mapping in the twisted multilayer WSe_2_ specimen, showing the strength and direction of the IP polarization within a selected Moiré unit cell. The long diagonal of the unit cell is highlighted, along with two high‐symmetry points, marked by a triangle and a inverted triangle, respectively. b) Plot of the IP polarization component projected onto the unit cell vector along the Moiré diagonal, showing variations in magnitude across the unit cell.

**Figure** **4**a shows the measured IP polarization P_∥_, calculated from the IP electric field ${\vec{E}}_{∥}$ by multiplying with the permittivity of vacuum (ɛ_0_) and the electric susceptibility χ_*e*_. Since polarization is directly proportional to the electric field, the IP polarization exhibits a similar structure. Two major vortices at 1/3 and 2/3 along the diagonal of the Moiré supercell, with an additional smaller vortex located at the corners. Figure 4b displays the IP polarization values along the major axis, highlighted by the dashed white line in Figure 4a. The IP polarization displays an oscillatory behavior as its direction changes along the diagonal to form the vortex structure. At the core of the vortices (denoted by triangles), the IP polarization is zero, in agreement with previous predictions of Moiré‐induced polarization textures.^[^ ^26^, ^27^ ^]^

These observations align with the findings of Magorrian et al.,^[^ ^28^ ^]^ which describe how Moiré‐induced lattice reconstruction generates ferroelectric potentials, modulating local polarization and electric fields. Additionally, this framework predicts that stacking‐induced reconstructions introduce nanoscale strain fields, which significantly influence polarization and electrostatic potential variations. Building on this framework, we extend our investigation to examine the role of localized strain in shaping the IP vortex‐like electric field textures.

### 2.2 Nanoscale Strain Mapping in Twisted Multilayer WSe_2_ Superlattices

To ascertain the correlation between vortex‐like electric field textures and local nanoscale strain fields, we applied an extended version of geometrical phase analysis (GPA).^[^ ^29^ ^]^ This adaptive GPA algorithm overcomes limitations of conventional^[^ ^30^ ^]^ GPA by addressing wave vector deviations amplified by the Moiré pattern. By dynamically adjusting reference wave vectors and calculating local phase gradients, the method enables accurate strain mapping, even for small‐angle Moiré structures. GPA was performed on the HRTEM image in Figure 1b, covering both the stripe region and surroundings areas to compare strain distribution across different stacking configurations.

**Figure 5:**
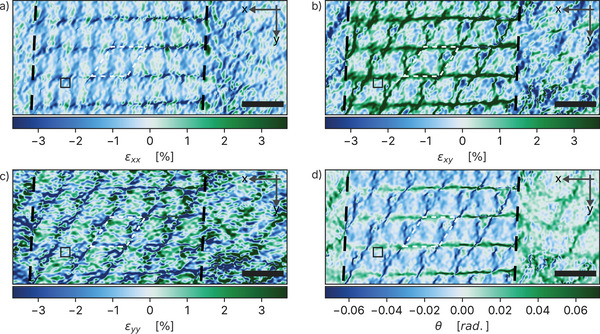
Nanoscale strain fields in twisted multilayer WSe_2_. Strain fields were calculated from the HRTEM micrograph in Figure 1b using Geometric Phase Analysis method as implemented in the pyGPA Python package. a–c) Maps of compressive (blue) and tensile (green) strain in the *x*‐direction (ɛ_*xx*_), shear strain (ɛ_*xy*_), and *y*‐direction (ɛ_*yy*_). d) Local rigid rotational misalignment (θ) in radians. All values are evaluated with respect to the reference region denoted by the solid black square. Scale bars: 10 nm in all panels.

**Figure** **5** displays the three strain components, ɛ_*xx*_, ɛ_*yy*_, ɛ_*xy*_, along with the rigid rotation angle θ, across the specimen's triple‐stacked stripe and the surrounding double‐stacked regions. All strain components exhibit a periodic pattern, which closely matches the vortex‐like electric field textures observed in the same region. Among these, the shear strain component ɛ_*xy*_ (Figure 5b) reveals the most distinct periodic modulation with features intricately linked to the underlying Moiré lattice. This periodic shear strain pattern is observed only within the stripe region, which is marked by dashed black lines in the figure, and vanishes outside this area. Notably, the tensile strain lines (green) in the ɛ_*xy*_ map form a regular network whose spacing matches the Moiré superlattice unit cell. Furthermore, the unit cell of this tensile strain pattern coincides with the corresponding features in the parallel electric field map (Figure 3b), which encompasses two vortices per unit cell.

Additionally, a spatial correlation emerges between strain distribution and electric field intensity. As seen in Figure 3, the electric field weakens toward the rightmost part of the triple‐stacked stripe. This behavior is mirrored in the ɛ_*xy*_ strain field map, where the tensile strain lines intersect, forming distinctive fork‐like structures (particularly prominent on the right side of the map). Within these forks, regions of compressive strain (blue) reach values as low as −3%, while the tensile strain along the green lines peaks at +3.5%, indicating strong modulation of the shear strain component. Interestingly, these crossing points of tensile strain lines coincide with the locations of the smaller electric field vortices. The magnitude of this strain, reaching up to ±3.5%, is consistent with values reported in Moiré superlattices of twisted TMDs and arises intrinsically from interlayer adhesion and Moiré‐driven lattice reconstruction. In the absence of externally applied strain, the system relaxes into energetically favorable stacking domains separated by strained dislocation networks, as also observed in bilayer TMDs.^[^ ^31^, ^32^ ^]^

Furthermore, from all strain components shown in Figure 5, the tensile strain patterns weaken towards the left and right sides of the map before eventually disappearing, a behavior that is consistently reproduced in the electric field maps of Figure 3. These changes occur across the dashed black lines, which delimit the triple‐stacked stripe. Outside this region, in the surrounding double‐stacked zones (8 + 2 ML), the strain fields become significantly less structured, and no periodic modulation or vortex‐like features are observed, despite the presence of Moiré contrast. This confirms that both strain modulations and polarization vortices are highly localized to the triple‐stacked geometry. The strong correlation between the Moiré pattern, the vortex‐like electric field textures, and the strain field maps unambiguously establishes that the formation of vortex‐like textures is driven by strain fields.

To further support this conclusion, we analyzed the full area surrounding the triple‐stacked stripe, including the adjacent double‐stacked zones formed by twist angles of 1.5°  (A + B), 2.3°  (A + C), and 0.7°  (B + C). As shown in Section S3 (Supporting Information), these regions exhibit no periodic strain modulation or vortex‐like electric field textures, despite the presence of Moiré contrast.

## 3 Discussion

Many qualitative features of Moiré‐induced polarization in bilayers appear in multilayers as well, but our twisted multilayer WSe_2_ system exhibits distinct polarization vortices with a uniform vorticity, in contrast to the alternating meron‐antimeron textures predicted for twisted bilayers.^[^ ^26^ ^]^ Theoretical models successfully describing bilayer polarization textures provide a natural starting point for understanding multilayers. In bilayer systems, two main factors are essential: i) lattice relaxation and reconstruction due to competing interlayer stacking energies, and ii) the resulting electrostatic potential landscape, which gives rise to periodic polarization domains.^[^ ^28^ ^]^ In these systems, Moiré‐induced lattice reconstruction generates regions of local 3R‐like and 2H‐like stacking, leading to an alternating polarization texture, where 3R stacking is polar while 2H remains non‐polar.

In contrast, the triple‐stacked stripe in our multilayer WSe_2_ system deviates from this expected alternation, we observe a single‐handedness of polarization vortices across the Moiré unit cell indicating a broken global inversion symmetry. This suggests a fundamental difference in symmetry and interlayer coupling compared to bilayers. A key factor is the presence of a third stacked flake in the triple‐stacked region forming a trilayer configuration that introduces additional symmetry breaking and enhances stabilizing a single rotational sense throughout the Moiré supercell. This observation suggests that the multilayer adopts a specific stacking sequence, likely rhombohedral‐like (3R) stacking, which enhances interlayer coupling. The effect of stacking order on the stabilization of chiral polarization vortices is a critical distinction between bilayers and multilayers. This observation aligns with recent theoretical models^[^ ^26^ ^]^ in bilayer systems, where strain acts in concert with stacking‐induced symmetry breaking to generate localized polarization textures, our result suggest this cooperative mechanism extends to multilayers as well. A more detailed theoretical analysis will be required to fully capture the mechanisms behind the directional alignment of polarization vortices observed in the triple‐stacked configuration.

Multilayer stacking also enhances out‐of‐plane polarization, which in turn strengthens in‐plane polar responses.^[^ ^33^, ^34^ ^]^ For instance, in trilayer TMDs, rhombohedral stacking is known to produce a larger out‐of‐plane dipole moment than bilayer 3R or bilayer 2H. In our Moiré system, this means that local rhombohedral‐like domains may carry a stronger vertical polarization than any bilayer domain would. This interfacial coupling locks domain configurations across layers, effectively coupling polarization through the thickness, increasing the energy cost of domain variations, and stabilizing the observed uniform vorticity. The presence of additional stacked monolayers thus constrains the polarization textures more tightly than in bilayers, influencing the formation of chiral polarization textures at the Moiré scale.

Our results also emphasize the critical role of nanoscale strain in enabling the observed Moiré polarization vortices. Through 4D‐STEM and EMPAD‐based diffraction analysis, we demonstrate that strain variations are intimately connected to localized electric field vortices. Geometrical phase analysis (GPA) mapping of strain fields reveals that shear strain components (ɛ_*xy*_) form periodic networks matching the Moiré unit cell, directly influencing the formation and periodicity of in‐plane polarization textures. This observation provides new insight into how strain fields modulate polarization at the atomic scale. In bilayer models, strain naturally arises due to lattice relaxation at the Moiré scale, but in a multilayer system, strain may not develop uniformly unless explicitly present. Our findings confirm that vortex formation is strain‐driven, emerging from the intrinsic strain modulations imposed by Moiré lattice reconstruction. The observed spatial correlation between alternating tensile and compressive strain and electric field vortex intensity highlights the role of strain as a symmetry‐breaking field that mediates the formation and alignment of in‐plane polarization textures. This suggests that strain engineering could serve as a tool to manipulate Moiré‐induced polarization textures, opening new possibilities for controlled domain architectures.

The emergence of uniform chiral polarization textures in twisted multilayer WSe_2_ has significant implications. The presence of a non‐zero net topological polarization charge per Moiré cell suggests that domain interactions, such as merging or termination at sample edges, could exhibit nontrivial electrostatic and topological behaviors. This also opens pathways for strain‐controlled nanoscale ferroelectric devices, where chirality in domain structures could be harnessed for functional applications. One immediate implication is in chiral domain wall dynamics in 2D van der Waals materials. Unlike bilayers, where alternating vortex textures ensure zero net chirality, our multilayer system forms globally chiral ferroelectric structures, which may interact uniquely with external electric fields. Such structures could enable nonreciprocal electronic or optical responses, relevant for optoelectronic applications such as circularly polarized second harmonic generation (SHG).^[^ ^35^ ^]^ Additionally, the strong interlayer coupling in the multilayer Moiré system suggests that domain switching and polarization control can be more stable and tunable than in bilayers, providing potential benefits for low‐power electronics and neuromorphic computing.

## 4 Conclusion and Outlook

This study demonstrates that strain‐induced polarization vortices in twisted multilayer WSe_2_ represent a new class of Moiré‐driven polarization textures, distinct from previously studied bilayer behaviors. The emergence of single‐handedness polarization vortices, enabled by interlayer coupling and stacking symmetry breaking, marks a significant departure from the alternating meron‐antimeron textures observed in bilayer systems.

Furthermore, strain mapping and polarization analysis confirm that nanoscale strain plays a decisive role in vortex formation, reinforcing the need to consider both strain and stacking configurations in the design of Moiré‐engineered polarization structures. These findings extend theoretical predictions on strain‐engineered polarization landscapes in twisted TMD bilayers to multilayer systems and reveal previously unrecognized form of chiral polarization order. Beyond validating these effects, this work provides an experimental foundation for the development of theoretical models describing strain‐tunable polarization textures in multilayer Moiré superlattices.

Looking ahead, these insights open new directions for Moiré‐tunable polarization control, suggesting that stacking order, strain fields, and external perturbations could be leveraged to control polarization textures and chirality in a programmable way. This tunability paves the way for next‐generation applications in topological excitations, low‐power electronics, and quantum materials, where polarization and chirality are key functional parameters.

## 5 Experimental Section

### Sample Preparation

The twisted multilayer WSe_2_ was fabricated by mechanically exfoliating bulk flakes using the scotch tape method. To obtain thin flakes, the material was cleaved multiple times before transferring to polydimethylsiloxane (PDMS) stamps, where further exfoliation between PDMS stamps was performed. After each exfoliation step, the stamps were inspected under an optical microscope to identify sufficiently thin material, which was then used for stamping. The selected flakes were transferred onto a polyvinyl alcohol (PVA), coated silicon substrate (1 cm by 1 cm) following the process outlined by Köster et al.^[^ ^36^ ^]^ To introduce a controlled twist angle, individual flakes were sequentially stamped using a dry viscoelastic transfer method, adjusting the rotation between layers during alignment under an optical microscope.^[^ ^37^ ^]^ After transfer, a lacey‐carbon TEM grid was brought into contact with the flake, with its active side facing the specimen. To promote adhesion between the flake and the TEM grid, the sample was wetted with isopropyl alcohol (IPA) and left to dry. The capillary force from IPA evaporation pulled the carbon foil into contact with the flake.^[^ ^36^ ^]^ Once the IPA had fully evaporated, the excess PVA was removed, and the sample was immersed in deionized water to dissolve the remaining PVA, allowing the TEM grid to detach and float freely. Finally, the sample was rinsed in deionized water and left to dry before proceeding with TEM inspection.

### Thickness Determination Via EELS

Electron energy‐loss spectroscopy (EELS) was used to quantify the thickness variations across the specimen. Thickness measurements were performed by acquiring EELS spectra at multiple locations and computing the local thickness using the Log–Log method in the Digital Micrograph software suite, following established quantification techniques.^[^ ^38^ ^]^ The thickness map was obtained by acquiring an EELS spectrum at each pixel, followed by thickness computation using the Log‐Log method in the Digital Micrograph software suite. This method was based on well‐established thickness quantification techniques described in ref. [38]. The thickness *t* was obtained from the inelastic mean free path (IMFP) using the relation:

(1) $$\frac{t}{\lambda }=ln\left(\frac{I_{0}}{I_{t}}\right)$$

where *I*_0_ is the zero‐loss peak intensity, *I*_*t*_ is the total spectral intensity, and λ is the IMFP, which depends on the material and electron energy. This method allows accurate estimation of thickness in units of IMFP, which is then converted to absolute thickness based on reference values for WSe_2_. The results confirmed distinct thickness variations across the sample, corresponding to eight monolayers (ML) in the darker region, 10 ML in the brighter region, and 12 ML in the narrow strip.

### 4D‐STEM Measurements

4D‐STEM analyses were performed with the EMPAD detector in a Titan Cubed microscope operated at 300 kV. To ensure the required spatial resolution for mapping polarization fields and local lattice distortions at the nanoscale, the electron probe size was carefully selected to be smaller than the spatial extent of the Moiré features, while its full‐width at half maximum (FWHM) remains larger than the interatomic distances within the crystal. The probe FWHM was estimated to be 0.8 nm which with a scan step size of 0.43 nm provides full coverage of the scan region. Acquisition settings on the EMPAD were set as follows: scale set to 1, scan size 128 × 128 real space probe positions, dwell time of 2 ms. The microscope was operated in Probe‐L STEM mode with ‘free ctrl’ selected such that a convergence angle α of 3.39 mrad could be selected with the C2 aperture of 50μm inserted. Furthermore, the spot size index was set to eight, the camera length to 1150 mm and the magnification to 1.8M×. These settings result in a dataset of 128 × 128 × 128 × 128 pixels, which was then analyzed as described in Section S2 (Supporting Information).

### Center of Mass Calculation

To quantify local electrostatic potential variations, shifts in the center of mass (CoM) of the bright‐field disk from 4D‐STEM diffraction patterns were analyzed. The CoM at each probe position **r** = (*x*, *y*) was computed as:

(2) $$k_{\text{CoM}}\left(r\right)=\left(k_{x,\text{CoM}}\left(x,y\right),k_{y,\text{CoM}}\left(x,y\right)\right)=\frac{\sum_{i}k_{i}I_{i}\left(r\right)}{\sum_{i}I\left(k_{i}\right)}$$

where *I*_*i*_(**r**) represents the intensity of the *i*‐th pixel in the diffraction pattern, associated with a momentum transfer **k**_*i*_, and the sum is carried over all the recorded pixels within the bright‐field disk. This method ensures that deflections in the electron beam caused by internal electric fields are captured, allowing direct mapping of IP polarization fields. Prior to CoM extraction, rigid beam shifts were corrected using cross‐correlation techniques (See Section S3, Supporting Information, for details.). During the analysis Python libraries rosettasciio, numpy, scipy, and matplotlib were used extensively.^[^ ^39^, ^40^, ^41^, ^42^ ^]^

### Geometrical Phase Analysis

Strain analysis using geometrical phase analysis was performed with the open source Python package  pyGPA . The package contains additional functions that allow for more accurate analysis on small‐angle Moiré as it optimizes over a number of reference vectors close to those vectors corresponding to the atomic lattice.

## Conflict of Interest

The authors declare no conflict of interest.

## Author Contributions

J.J.M.S. and A.B. prepared the twisted multilayer WSe_2_ specimen. J.J.M.S. performed the 4D‐STEM experiments, collected the data, and developed the computational tools for 4D‐STEM analysis. J.J.M.S. also conducted the data analysis and interpretation of 4D STEM measurements, with input from S.C.‐B. J.J.M.S. and S.C.‐B. co‐wrote the initial draft of the manuscript, with input and revisions from all authors. The final version of the manuscript was reviewed and edited by S.C.‐B., J.J.M.S., and A.B. S.C.‐B. supervised all aspects of the project.

## Supporting information

Supporting Information

## Data Availability

The data that support the findings of this study are available from the corresponding author upon reasonable request.
