# Supplementary material for: Strain‐Induced Moiré Polarization Vortices in Twisted‐Multilayer WSe2
Source: Small. 2025 May 20;21(40):2503363. doi: 10.1002/smll.202503363 (PMC12508720; doi:10.1002/smll.202503363)
Supplement: Supplementary file 1 — Supporting Information [file SMLL-21-2503363-s001.pdf]

NANO · MICRO
small

Supporting Information

for *Small*, DOI 10.1002/smll.202503363

Strain-Induced Moiré Polarization Vortices in Twisted-Multilayer WSe_2

*Jeroen J.M. Sangers, Abel Brokkelkamp and Sonia Conesa-Boj**

Strain-Induced Moiré Polarization Vortices in Twisted-Multilayer WSe_2: Supplementary Information

Jeroen JM Sangers[1], Abel Brokkelkamp[1], Sonia Conesa-Boj[1,*]

[1]Kavli Institute of Nanoscience, Delft University of Technology, 2628 CJ, Delft, The Netherlands

*Corresponding author. Email: s.conesaboj@tudelft.nl

Contents

S1 Fast Fourier Transform (FFT) Analysis **2**

S2 4D-STEM Data Processing **3**

S3 Additional Twisted Multilayer System **5**

S1 Fast Fourier Transform (FFT) Analysis

Fast Fourier Transform (FFT) analysis (Fig. S1) quantitatively confirms the rotational misalignments in the twisted WSe_2 multilayers. The two bottom flakes (8 ML and 10 ML) exhibit a twist angle of 1.5° relative to each other, while the stripe region shows additional misalignments of 2.3° relative to the bottommost layer and 1.0° relative to the adjacent 10 ML region.

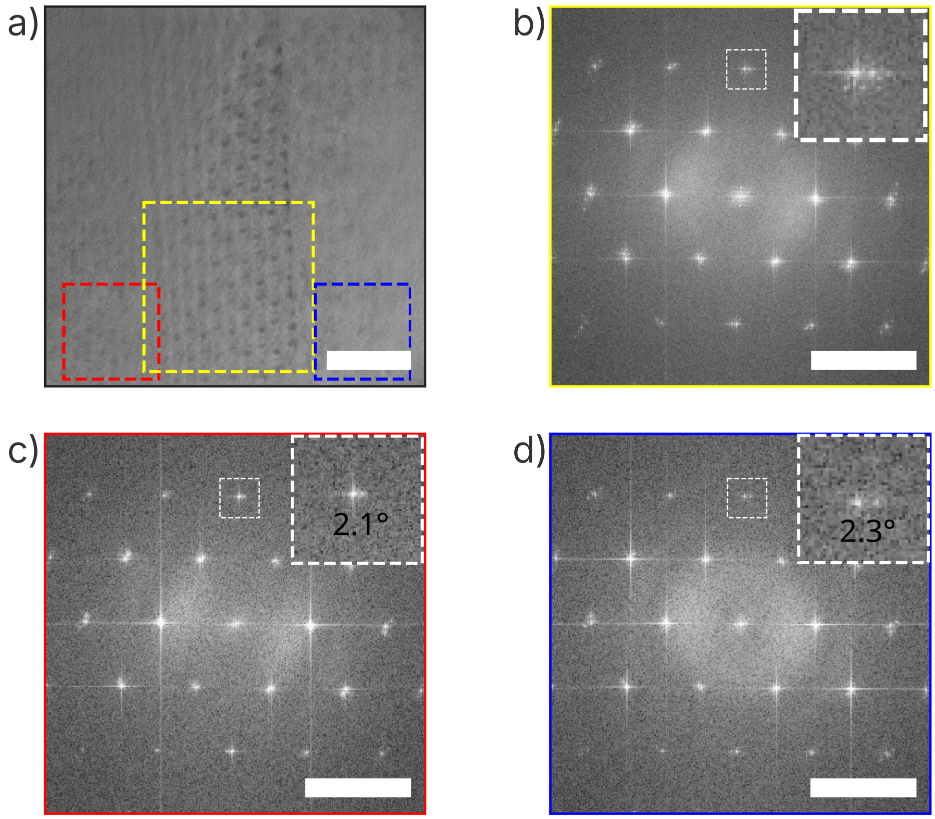

Figure S1: Fast Fourier Transform (FFT) Analysis of Rotational Misalignments. FFT analysis performed on different regions of the specimen to quantify rotational misalignments and confirm Moiré periodicity. **a)** High-resolution transmission electron microscopy (HRTEM) image of the twisted multilayer WSe_2 sample. The colored dashed squares indicate regions where FFT was performed: yellow (stripe region), red and blue (outside the stripe in the 10 ML region, as referenced in Fig. 1 of the main text). **b)** FFT of the yellow-marked region, located inside the stripe, where additional satellite spots indicate Moiré periodicity due to the local twist. **c)** FFT corresponding to the red-marked region, showing a distinct diffraction pattern representative of the 10 ML area. **d)** FFT of the blue-marked region, another area in the 10 ML region outside the stripe, displaying a similar diffraction pattern to (c). Scale bars are 25 nm and 5 nm^{-1}.

S2 4D-STEM Data Processing

4D-STEM measurements allow for the recording of a reciprocal space electron diffraction micrograph (see Fig. S2) for every probe position of the electron beam during its rasterization across the specimen. Such a dataset is characterized by a recorded electron intensity for two real-space coordinates, set by the probe positions during the scan, and two reciprocal-space coordinates given by the electron diffractogram.

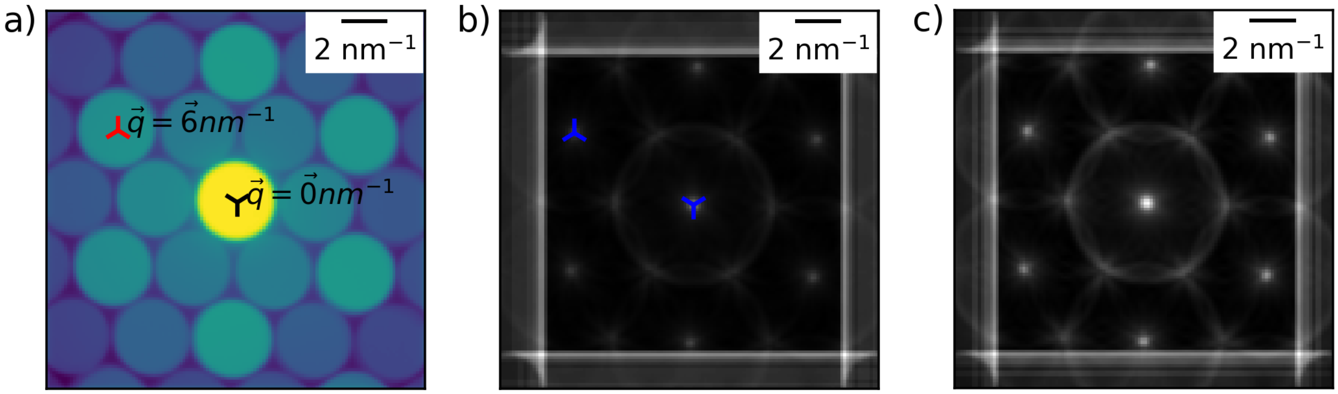

Figure S2: a) Position-averaged electron diffractogram showing the average of all recorded diffractograms used for the calibration of the EMPAD detector measurements. **b)** Result of convolution with optimal kernel before correction of the rigid shift. Highlighted with blue markers are the centers of the disks used for calibration. **c)** Result of convolution with optimal kernel after correction of rigid shift.

A 4D-STEM dataset encodes a wealth of information and can be used to achieve similar measurements as those obtained with various traditional STEM detectors, such as a bright- or dark-field detector whose outer collection semi-angle is less than the maximum set during acquisition of the 4D-STEM dataset. Results from segmented annular detectors, such as those originally used for CoM analyses, can not only be reproduced but also improved upon by careful processing of the 4D-STEM data acquisition. Whereas traditional segmented detectors analyze the difference in electron intensity between two opposing segments to compute a beam deflection value, a 4D-STEM dataset is analyzed by taking a weighted sum of the intensity value per pixel; akin to computing the CoM for a set of discrete masses.

In 4D-STEM measurements, before computing the CoM of the bright-field disk, a series of corrections need to be applied to the dataset to ensure the robustness of the extracted CoM signals. Firstly, the center coordinate of the bright field disk and a higher-order reflection need to be found to calibrate the reciprocal-space pixel size, to achieve this a in radius varying annular kernel was convolved with an electron diffractogram. The optimum was considered 'found' when the peak intensity was maximised. The result of such an analysis is displayed in Fig. S2b. This creates an image with bright pixels at diffraction disk centers that is used to calibrate all diffractograms.

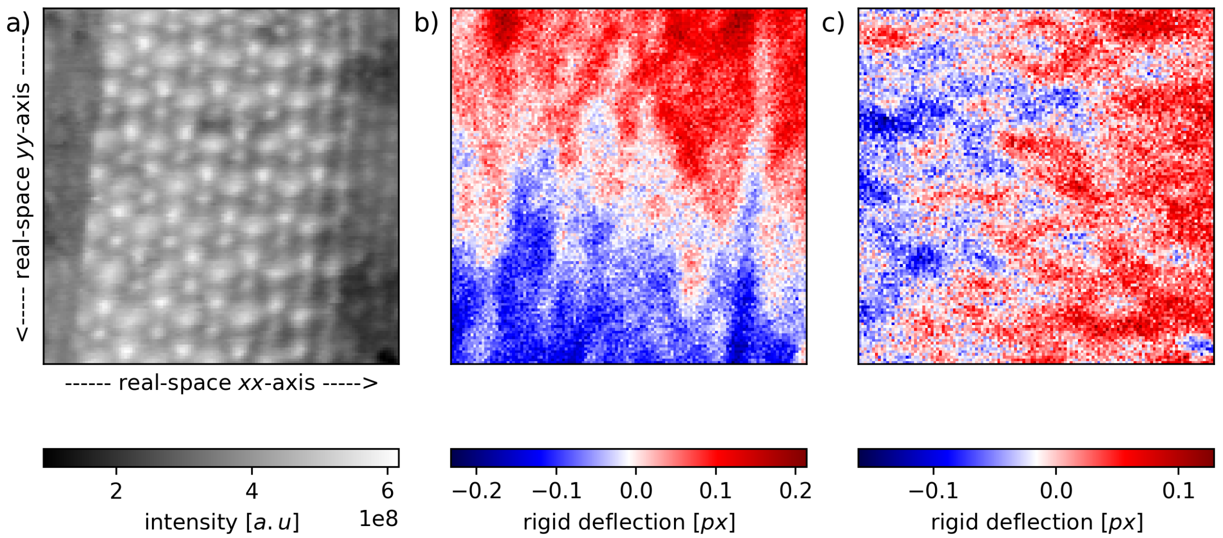

Figure S3: a) Annular Dark Field (ADF) image obtained by masking and integrating over the 4D-STEM dataset. **b)** Computed rigid shift (deflection) in the x-direction of the entire diffractogram over the sensor w.r.t. a neutral diffractogram. **c)** Same as **b** in the y-direction.

Depending on the relation between the electron probe size and the extent of the potential of the imaged feature, there are two intertwined but distinct effects that are observed. When the extent of the effective potential is much larger than the width of the electron probe, a rigid shit of the entire diffractogram with respect to the neutral position is observed [1, 2]. If instead the probe's width exceeds that of the imaged feature, as is the case here, an intensity redistribution within the bright-field (BF) disk is observed.

In the 4D-STEM method, deflections in the central CBED disks are caused by interactions with features larger than the probe FWHM, whereas electric fields in the sample cause a redistribution of intensity within the central disk [3, 1]. This effect is highlighted in Fig. S3b, where the edge of the strip and its change in thickness clearly shows up with a rigid deflection of the beam into the thicker region. To disentangle influences from both mechanisms, the rigid shift is corrected for prior to the CoM analysis of the BF disk.

The correction for the rigid shift is performed by first convolving the diffractograms with the two Sobel matrices to compute the edges of the disks; this desensitizes the correction of the rigid shift to any redistribution of intensity within the disks. All electron diffractograms are now shifted by the inverse of the offset of the brightest pixel in the computed cross-correlation power spectrum (CCPS) from the center. For sub-pixel accuracy of the correction, a two-dimensional Gaussian is fitted to the CCPS such that its center is equal to the rigid shift and its width can be used as a measure of uncertainty. This correction is performed for all diffractograms such that all BF disks have centers at the zero position of the detector.

Subsequently, the CoM is computed for every diffractogram, yielding two signals: one for CoM in the x-direction and one in the y-direction, both displayed in Fig. 2e-f of the main text, respectively. The CoM_y signal had to be inverted as the `rawFlipVertical` setting of the EMPAD was incorrectly set to `False` at the time of acquisition. The shifting of the diffractograms and the computation of the center-of-mass are performed using the `scipy.ndimage.shift` and `scipy.ndimage.centre_of_mass` functions respectively, both implemented in SciPy [4].

Finally, once the 4D-STEM dataset has been fully corrected and calibrated, it is possible to compute the parallel (in plane) component of the electric field/polarization from the measured CoM signals from Eq. (2) in the main manuscript. The vorticity of the electric field is then calculated by computing the curl of the resulting $E_{\parallel}$-field.

S3 Additional Twisted Multilayer System

To further emphasize the role of localized strain in enabling the formation of Moiré-induced polarization vortices, we examined two additional twisted regions from the same multilayer WSe_2 sample using both four–dimensional (4D)-STEM and conventional high-resolution TEM imaging.

The first analysis, described in the main text (Figures 2–5), includes the entire area surrounding the triple-stacked stripe (Figure S4). This region encompasses several double-stacked zones formed by different pairwise combinations of the two flakes: A + B (1.5°) and A + C′ (2.3°). While these zones exhibit Moiré contrast, they show no measurable vortex formation or periodic strain modulation.

Specifically, the (A + C′) region corresponds to stacking between flakes A and C′, located on the upper side of the triple-stacked stripe, with a relative twist angle of approximately 2.3°. We also considered a second region (A + C′), positioned further in the same direction, features a smaller twist angle of approximately 0.7° (see Figure S5). Both are double-stacked zones with a total thickness of 10 monolayers (8 ML + 2 ML), and neither shows polarization vortices or significant periodic strain modulation. These regions can be spatially located in the overview image in Fig. 1a of the main text.

Extended Field of View Around the Triple-Stacked Stripe

To provide a direct comparison with the strain behavior observed in the triple-stacked stripe (A + B + C, discussed in the main text), we analyzed the surrounding double-stacked zones — A + B (1.5°) and A + C′ (2.3°) — captured in the same high-resolution TEM field of view. These regions are highlighted in Figure S4, which displays the full strain maps derived via geometric phase analysis (GPA). In the triple-stacked stripe, previously shown in Figure 5 of the main text, shear strain amplitudes ε_{xy} reach up to $\pm 3.5\%$, forming a well-defined Moiré-periodic modulation pattern. In contrast, the double-stacked regions exhibit significantly weaker and more irregular strain distributions: in the A + C′ zone (top-right of the figure), the shear strain components ε_{xx}

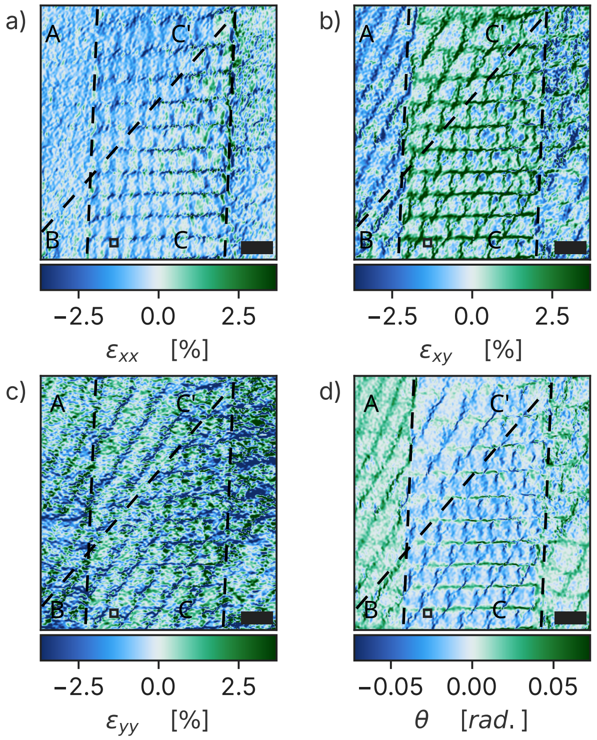

Figure S4: Strain mapping in the triple-stacked stripe region and surrounding double-stacked regions. a)-c) Maps of in-plane strain components: ε_{xx}) (tensile in green, compressive in blue), ε_{xy} (shear) and ε_{yy}. **d)** Local rigid rotation angle θ (in radians). The field of view includes the triple-stacked stripe and the adjacent double-stacked regions (A + B and A + C′). Dashed black lines denote the boundaries of the stripe, as well as the interfaces between the base flake (A) and the overlapping regions (B, C, and C′). Scale bars are 10 nm.

and ε_{yy} remain below $\pm$1.0%. This suggests that, although the twist angle is relatively large, the interlayer interaction in this bilayer region is insufficient to drive significant strain modulation.

In region A + B (bottom-left of the figure), the strain is even weaker: all components are typically below $\pm$0.3%. No periodic features are observed in this area, confirming the absence of Moiré-induced reconstruction.

A+C′ Region (0.7° Twist)

Figure S5 displays the strain and rotation fields derived using `pyGPA` package for the A + C′ region, which exhibits a twist angle of approximately 0.7°. Figure S5 (a-d) show the in-plane strain components ε_{xx}, ε_{xy}, and ε_{yy}, along the local rigid rotation angle (θ). The reference region used for GPA computation is indicated by a black box in each panel. Although weak, periodic contrast is observable in the strain maps—consistent with Moiré modulation at this small twist angle—the strain amplitudes are uniformly low and do not display the strong localization or symmetry breaking seen in the triple-stacked stripe. Specifically, the ε_{xx} and ε_{yy} not exceed $\pm$0.3%, and the shear strain

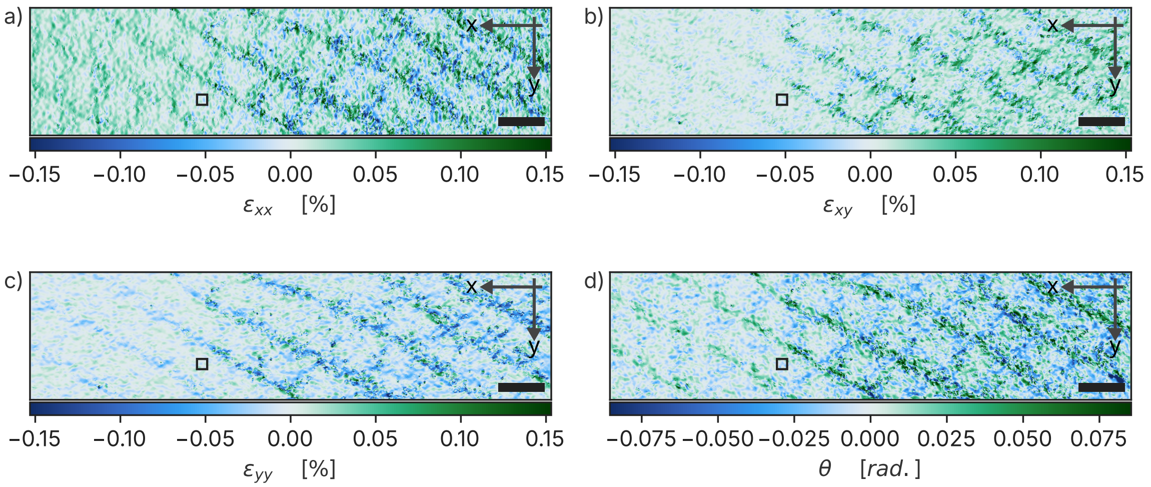

Figure S5: Strain in a a low-angle double-stacked region (A + C′, 0.7° twist) a-c Maps of the in-plane strain components: ε_{xx} (tensile in green, compressive in blue), ε_{xy} (shear), and ε_{yy}. **d** Map of the local rigid rotation angle θ (in radians). All values are evaluated relative to the underformed reference region denoted by the solid black box. Scale bars: 10 nm in all panels.

ε_{xy} stays within ±0.15%. These values are at least an order of magnitude smaller than those observed in the stripe region described in the main text (Figure 5), where strain modulations reached up to ±3.5% The lack of significant tensile or shear strain features, combined with the absence of dislocation networks, confirms that the material does not undergo strain-driven reconstruction in this configuration.

Analysis of the center–of-mass (CoM) redistribution signal was performed following the same methodology described in section S2 of the Supplementary Information. After correcting for the rigid shift, the CoM signal was extracted from the intensity distribution within the bright–field disk. The results are displayed in Figure S6, which presents the annular dark–field (ADF) image (panel a), CoM components along the x and y (panels **b** and **c**), and the CoM magnitude map (panel **d**).

The CoM signals spatially uniform and lack any noticeable periodicity. No clear modulation or vortex-like features are present in either direction, and the overall signal intensity is at least an order of magnitude weaker than in the triple-stacked stripe (main text, Fig. 5). These results confirm that the electric field texture in this low-angle, double-stacked region is featureless, and that the presence of a Moiré pattern alone—without significant local strain is insufficient to induce polarization vortices.

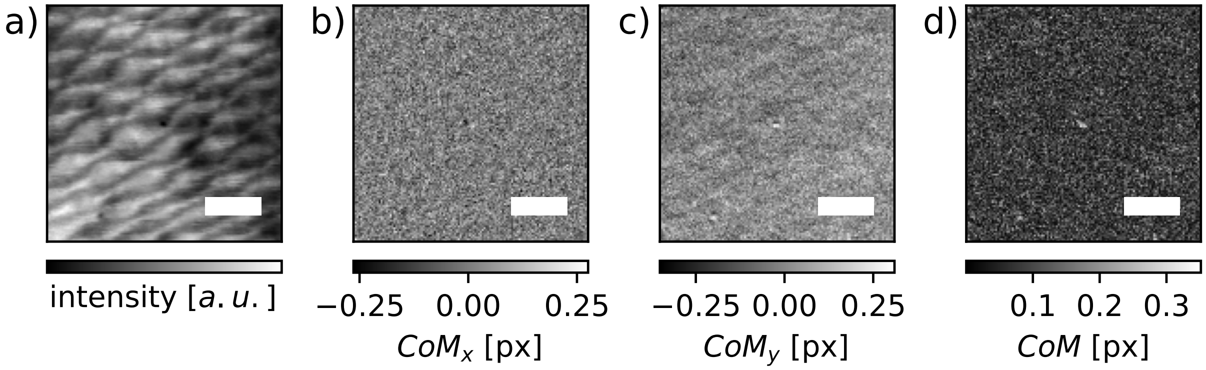

Figure S6: Center–of–mass (CoM) analysis in in a low-angle double-stacked region (A + C′, 0.7° twist)

a) Reconstructed ADF image generated from the 4D-STEM dataset using a virtual annular mask. The image reveals the Moiré contrast arising in the double–stacked region. **b)-c)** CoM redistribution within the bright-field disk along the x– and y–directions, respectively. **d)** Magnitude of the CoM vector, highlighting the overall strength of electric field variation. All scale bars are 10 nm

References

[1] Cao, M. C. *et al.* Theory and practice of electron diffraction from single atoms and extended objects using an EMPAD. *Microscopy* **67**, i150–i161 (2018). URL `https://academic.oup.com/jmicro/article/67/suppl_1/i150/4835603`.

[2] Clark, L. *et al.* Probing the limits of the rigid-intensity-shift model in differential-phase-contrast scanning transmission electron microscopy. *Physical Review A* **97**, 043843 (2018). URL `https://link.aps.org/doi/10.1103/PhysRevA.97.043843`.

[3] Lazić, I., Bosch, E. G. & Lazar, S. Phase contrast STEM for thin samples: Integrated differential phase contrast. *Ultramicroscopy* **160**, 265–280 (2016). URL `https://linkinghub.elsevier.com/retrieve/pii/S0304399115300449`.

[4] Virtanen, P. *et al.* SciPy 1.0: Fundamental Algorithms for Scientific Computing in Python. *Nature Methods* **17**, 261–272 (2020).
